# Non-specific Effects of Vaccines Illustrated Through the BCG Example: From Observations to Demonstrations

**DOI:** 10.3389/fimmu.2018.02869

**Published:** 2018-12-04

**Authors:** Deeva Uthayakumar, Simon Paris, Ludivine Chapat, Ludovic Freyburger, Hervé Poulet, Karelle De Luca

**Affiliations:** ^1^R&D Lyon, Boehringer Ingelheim Animal Health, Saint Priest, France; ^2^Département Biologie, Faculté des Sciences et Techniques, Université Claude Bernard Lyon 1, Villeurbanne, France; ^3^Faculté de Médecine Jacques Lisfranc, Université Jean Monnet, Universités de Lyon, Saint-Etienne, France; ^4^Agressions Pulmonaires et Circulatoires dans le Sepsis (APCSE), VetAgro Sup, Marcy l'Etoile, France

**Keywords:** BCG, vaccines, epidemiology, non-specific effects, trained immunity, epigenetics

## Abstract

Epidemiological studies regarding many successful vaccines suggest that vaccination may lead to a reduction in child mortality and morbidity worldwide, on a grander scale than is attributable to protection against the specific target diseases of these vaccines. These non-specific effects (NSEs) of the Bacille Calmette-Guérin (BCG) vaccine, for instance, implicate adaptive and innate immune mechanisms, with recent evidence suggesting that trained immunity might be a key instrument at play. Collectively referring to the memory-like characteristics of innate immune cells, trained immunity stems from epigenetic reprogramming that these innate immune cells undergo following exposure to a primary stimulus like BCG. The epigenetic changes subsequently regulate cytokine production and cell metabolism and in turn, epigenetic changes are regulated by these effects. Novel -omics technologies, combined with *in vitro* models for trained immunity and other immunological techniques, identify the biological pathways within innate cells that enable training by BCG. Future research should aim to identify biomarkers for vaccine heterologous effects, such that they can be applied to epidemiological studies. Linking biological mechanisms to the reduction in all-cause mortality observed in epidemiological studies will strengthen the evidence in favor of vaccine NSEs. The universal acceptance of these NSEs would demand a re-evaluation of current vaccination policies, such as the childhood vaccination recommendations by the World Health Organization, in order to produce the maximum impact on childhood mortality.

## Introduction

For over two centuries, vaccines have risen to their place amongst the most significant public health interventions in human history; the introduction of notable vaccines such as the smallpox vaccine, Bacille Calmette-Guérin (BCG), Oral Polio Vaccine (OPV), and measles-containing vaccines have reduced morbidity and mortality worldwide. Smallpox in humans and rinderpest in cattle have been eradicated, and polio is next in line (1).

For many of these vaccines, the exact correlates of protection are still unknown and science has only recently begun to elucidate their biological interactions with the human immune system (2). There are numerous observations suggesting that the significant public health impact of these vaccines goes beyond simply offering protection against their respective target diseases, especially noted in live vaccines. For example, implementation of measles-containing vaccines in various studies reduces all-cause mortality ranging from 30 to 86%; this depression far exceeds the mortality that is induced by measles illness alone, and is postulated to also reduce bacterial carriage related to sepsis and pneumonia (3). Similarly, the OPV vaccination campaign that was launched in 1998 in Guinea-Bissau was linked to a decreased mortality rate ratio in children under the age of 5, independent of the vaccine efficacy against polio (4). Different heterologous effects due to vaccination, such as protection against unrelated pathogens, anti-tumor properties, and an all-around drop in child mortality have been coined as “off-target” or non-specific effects (NSEs). The NSEs of BCG have been explored for over half of a century now, and BCG has thus become a model of interest for studying such heterologous effects.

Use of the live attenuated BCG vaccine to protect children against severe forms of tuberculosis (TB) became widespread since 1924; a virulent *Mycobacterium bovis* strain was passaged 230 times from 1908 to 1921 on a glycerin, beef bile, and potato medium to obtain BCG (5). The vaccine strain was distributed to different producers (each implementing their own manufacturing procedures) such that many BCG vaccine strains exist today, with varying degrees of attenuation and efficacy against TB (5). The different strains of BCG, and their corresponding diverse target populations, make it difficult to extrapolate epidemiological observations made in one setting toward a more global perspective. Nevertheless, there is growing evidence that supports the presence of NSEs, in both clinical and animal studies.

While pioneer epidemiological studies observe a remarkable reduction in all-cause mortality, the diversity in study designs, inconsistent results, and an inability to firmly correlate BCG NSEs with these results decreases their utility. Similarly, *in vitro* models and animal studies couple possible mechanisms to these puzzling NSEs but fail to demonstrate relevancy in a clinical setting. With novel technologies emerges new scientific evidence regarding NSEs; it is important to strengthen this knowledge and identify new biomarkers that can be used in the clinical setting. The union between molecular biology techniques and epidemiological observations represents the future of vaccine NSE research. Together, these two methodologies could give rise to concrete data that can influence current vaccination policies and optimize existing vaccination schedules.

## Review of the Past: Epidemiological Observations of BCG Heterologous Effects

Until recent years, the strongest evidence supporting BCG NSEs in humans came from the randomized trials in Guinea-Bissau (2002–2008) (Table 1). Due to faulty randomization, the early trial with 105 participants ended in 2004, and then restarted as the main Guinea-Bissau study (18). The main study, like the early study, compared low-birth-weight children who were vaccinated at birth to those who delayed vaccination until 6 weeks of age (19). The investigation, with a sample size of 2,320 infants, revealed that BCG administration as early as possible to newborns of low birth weight corresponds to a reduction in the neonatal mortality rate by over 40% in the first month following immunization, most probably linked to fortifying neonatal defense mechanisms against general septicemia and pneumonia (19, 20).

**Table 1:** Summary of epidemiological studies investigating BCG NSEs.

**Country (study period)**	**Sample size**	**Subject follow-up period**	**% Reduction in all-cause mortality**	**Risk of bias**	**References**
Canada (1933–1945)	609	6–14 years	6% (−32; 33)	Moderate	(6)
USA (1935)	3008	2 years	9% (−99; 59)	Moderate	(7)
USA (1941–1960)	451	Up to 13 years	58% (−35; 87)	Moderate	(8)
Benin (1983–1987)	294	4-36 months	32% (−23; 62)	High	(9)
Guinea-Bissau (1984–1996)	1657 + 695 + 4418	6-8 months	37% (−33; 70) 95% (54; 99) 44% (16; 63)	High	(10, 11)
India (1987–1989)	3072	12 months	40% (−97; 82)	High	(12)
Papua New Guinea (1989–1994)	3937	1-6 months	83% (66; 91)	High	(13)
Malawi (1995–1997)	751	8 months	55% (−23; 84)	High	(14)
Senegal (1996–1999)	4421	2 years	2% (−90; 50)	High	(15, 16)
India (1998–2002)	10274	6 months	56% (34; 71)	High	(17)
Guinea-Bissau (early: 2002–2004)	105	1 month	72% (−37; 94)	Low	(18, 19)
(main: 2004–2008)	2343	1 month	45% (11; 66)		

*The studies systematically reviewed by the SAGE were determined to be inconclusive, as they did not link the reduction in all-cause mortality with mechanisms of NSEs. Studies assessed as having a very high risk of bias are not included. The percentage of reduction in all-cause mortality consists in a 95% Confidence Interval (CI) further specified by (1-risk ratio). The classification of the risk of bias is as reviewed by the SAGE Working Group*.

Prior to this, quasi-randomized studies were confined only to the United States and Canada. A 1933 to 1947 study in Canada examined an indigenous population with high tuberculosis (TB) incidence (Table 1) (6). Six hundred and one individuals were observed over 6–14 years after being either vaccinated or not vaccinated within 10 days of birth. This study mainly explored the incidence of death from TB in vaccinated vs. unvaccinated individuals, but also measured death from all causes. There was an observed 6% reduction in the risk ratio (95% CI: −32–33) of all-cause mortality in vaccinated indigenous children vs. unvaccinated, a seemingly small yet significant impact (6). The risk ratio in this case pertains to the cumulative incidence of death occurring in a vaccinated group of individuals vs. an unvaccinated group; the smaller the ratio, the more beneficial vaccination is at reducing the risk of mortality (21). Likewise, from 1935 to 1938, over 3,000 indigenous peoples in the United States took part in a BCG clinical trial which demonstrated a 9% reduction in the risk ratio of death between the vaccinated vs. unvaccinated within a 2-year follow-up period (95% CI: −99–59) (Table 1) (7). The decline in mortality in both these cases was not large, and thus, difficult to draw conclusions from. In addition, observations in indigenous communities do not reflect the true diversity of the North American population. In the urban setting of Chicago 451 newborns from households with a TB history were monitored and a 58% reduction in the risk ratio in BCG recipients was observed compared to non-recipients when children were followed up to 13 years (95% CI: −35–87) (Table 1) (8). This percentage is much larger than those observed in the indigenous communities. However, some parameters varied between studies: the sample size, the follow-up period used and the prevalence of TB-related deaths.

Aside from these randomized trials, various observational studies were made from the mid-1900s to early 2000s, most notably in Guinea-Bissau, India, Malawi, Papua New Guinea, and Senegal (Table 1). These studies generally indicated a beneficial effect of BCG, though the demonstrated reduction in relative risks of all-cause mortality were extremely variable, ranging from 2 to 95%, not to mention that approximately half of these studies occurred in Guinea-Bissau (Table 1) (9–17). Despite some studies adjusting for age and gender, such as the Guinea-Bissau studies in the 1980s−1990s, it is difficult to base conclusions off of non-randomized conditions where too many confounders may be unaccounted for. Lastly, although these data suggest an effect in low income settings, no such observations are seen in high income settings (11).

None of the studies showed a difference in protective effects between males and females. The mean age of infants receiving the BCG vaccine also differed between studies, as young as 2 days old to as old as a year (11). Vaccination at earlier time points was associated to a more positive impact by NSEs and a greater reduction in all-cause mortality in non-randomized studies in Guinea-Bissau (1980s−1990s) and Bangladesh (1986–2001). The Guinea-Bissau randomized studies (2002–2008) also supported these observations by comparing low-birth-weight infants of two groups: those vaccinated at birth and those vaccinated at 6 weeks as part of the regular vaccination schedule. In the main study, the risk ratio of death for those vaccinated at birth vs. at 6 weeks was 55% (95% CI: 11–66) (19). Repeated studies, in diverse populations, are required to demonstrate reproducibility and increase confidence in the observations.

Due to the indirectness of many studies in measuring non-TB-related deaths and the large variability among study designs (such as patient follow-up period and national vaccination schedules), the Strategic Advisory Group of Experts on Immunization (SAGE) within the World Health Organization (WHO) has declared in their report that studies about NSEs of BCG (as well as all vaccines in general) are inconclusive (11, 22). After assessing bias using the Cochrane Collaboration tool for randomized studies and a similar tool called ROBINS-I for non-randomized studies, most of the studies supporting BCG NSEs had a high risk of bias, with many confounding factors such as gender, age, child nutritional status, socioeconomic status, and co-interventions or -morbidities (i.e. malaria treatment, impact of other vaccines) (Table 1) (11, 23)Despite the verdict, they do acknowledge that the potential for NSEs is present. Work done by immunologists and epidemiologists increasingly support the existence of vaccine heterologous effects. Compared to the inconclusive studies made to investigate NSEs in humans, studies in mice demonstrate biological plausibility for BCG-induced NSEs.

## Painting the Present: Demonstration of BCG NSEs Biological Plausibility

### Challenge Studies in Mice

As a benchmark model to investigate NSEs, the use of murine models to study BCG allowed the observations of heterologous protection against unrelated pathogens. For example, immunization of specific-pathogen free mice with BCG, or a purified protein derivative of the Mycobacterial antigen tuberculin, displayed an activated macrophage phenotype that produced elevated levels of reactive oxygen species to induce acquired immunity against systemic *Candida albicans* infection. Alleviation of the infection was seen through the reduced load of *Candida* in the spleen, kidneys, and liver, and over a 50% reduction of invasive *Candida* germ tubes (24, 25). Similarly, nude mice were protected from pulmonary schistosomules when challenged with the cercaria of *Schistosoma mansoni* (26). Challenge with other pathogens, including *Babesia* and *Plasmodium* infections, also demonstrated protective BCG NSEs in mice (27). These studies suggest that it is indeed possible to obtain immune protection against one pathogen due to prior insult from a dissimilar microbe. Presuming that BCG possesses a hidden capacity to induce broad immune responses, many mechanisms were proposed to support the biological plausibility of these observations and the quest for knowledge extended to human studies.

### Possible Mechanisms of Action

For many decades, NSEs have been demonstrated in murine models through primary stimulation with BCG, subsequent challenge with unrelated pathogens and comparing outcomes between vaccinated and unvaccinated animals. *In vivo* murine challenge models are versatile; monitoring immune responses and disease progression can be carried out not only via blood collection, but also via biopsies, bypassing the ethical limitations that human studies face. Only in the last few years have attempts been made to demonstrate mechanistic events responsible for NSEs in humans; with these efforts came a plethora of methods to identify human biomarkers, from cytokine responses to epigenetic indications.

**Figure 1:**
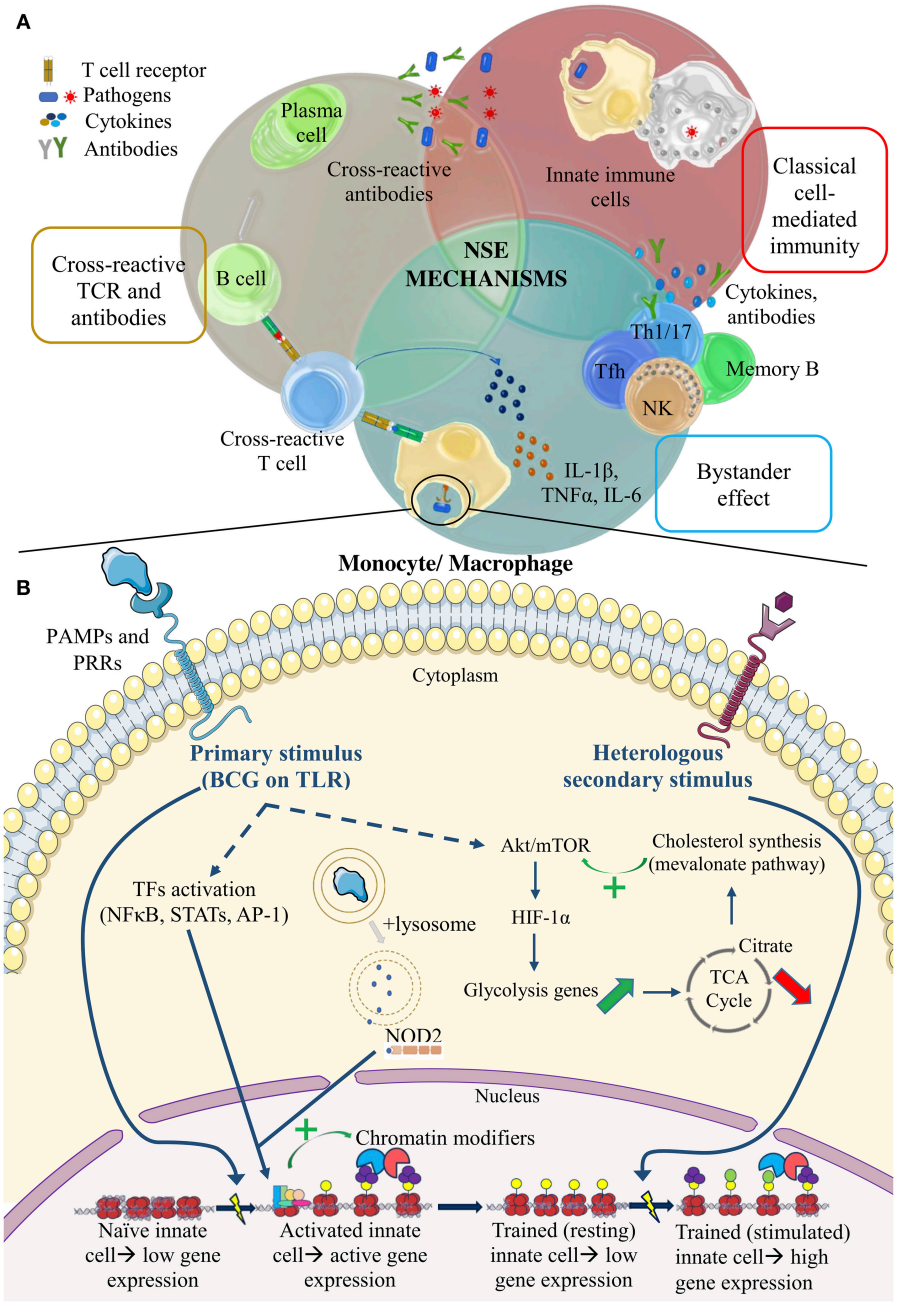
Summary of the potential mechanisms of vaccine NSEs. **(A)** The top half illustrates adaptive mechanisms. Cross-reactive TCRs and antibodies. Lymphocyte antigen receptors recognize similar epitopes from different antigens. Bystander effect. Bystander activation of pre-existing effector or memory cells occurs via changes in the cytokine environment. Classical cell-mediated immunity. Adaptive immune cells potentiate the non-specific activity of innate cells in classical cell-mediated immunity. **(B)** The bottom half illustrates pathways of trained immunity. Primary stimulus of BCG. PRR signaling leads to TF activation, which then recruits chromatin modifiers to genes of interest. This stimulus also activate the autophagy and NOD2 signaling pathway. Upregulation of the Akt/mTOR pathway alters metabolite levels that regulate chromatin-modifying enzymes. Heterologous secondary stimulus. Epigenetic changes within innate cells after training act as de novo enhancers to boost the immune response against a secondary challenge.

One possible NSE mechanism investigated is the cross-reactivity between vaccine antigens and antigens from unrelated vaccines or pathogens (Figure 1A). Despite different origins, some T- and B-cell epitopes may be shared between pathogens (1, 28). However, it is difficult for a relatively rare phenomenon like cross-reactivity to solely account for the extremely diverse heterologous effects seen with BCG. In addition, no particular BCG epitope can yet be connected to protection against non-Mycobacterial species.

An alternative hypothesis proposes that BCG enhances T helper 1 and 17 (Th1, Th17) cell polarization, generation of memory CD4+ T cells, Natural Killer (NK) cell memory, and the corresponding cytokine induction (1, 29, 30). A Dutch study on a small group of BCG-vaccinated young adults demonstrated that non-Mycobacterial stimuli were able to induce heterologous Th1 and Th17 cytokines, such as IFN-γ and IL-17, up to 1 year after receiving the vaccine. Enzyme-linked immunosorbent assays (ELISAs) were used to measure cytokine levels in the plasma (29). It seems that even these antigen-specific memory cells (i.e., memory T and NK cells) can undergo heterologous or “bystander” lymphocyte activation, since memory cells require less signals to be activated upon a second stimulus (Figure 1A). Observations in the Dutch study could be attributed to the bystander effect, where BCG components stimulate cytokine production and create a specific cytokine milieu that subsequently leads to existing polyclonal effector T cell (or NK cell) activation or production of antibodies (Abs) by memory B cells (Figure 1A). More recently, studies show that a general enhancement of antibody responses could be attributed to the special ability of live attenuated vaccines to promote the production of cytokines by innate cells that favor T follicular helper (Tfh) cell polarization in the germinal center of lymph nodes, promoting affinity maturation of B cells and B cell memory formation (31). In the case of BCG, RNA pathogen-associated molecular patterns (PAMPs), act as markers of microbial viability, and are sensed by Toll-like receptor 8 (TLR8) on monocytes and dendritic cells. Downstream signaling of this receptor-ligand interaction could lead to selective induction of the IL-12p40 component of the IL-12 cytokine and subsequent development of Tfh cells in the lymph node via upregulated IL-12-receptor signaling; killed vaccines do not produce this effect (31). To complement these *in vitro* findings, epidemiological studies identified that hypermorphic TLR8 polymorphisms enhance the protection induced by BCG. Thus, TLR8 could have a central role in the action of live attenuated vaccines in individuals to promote Tfh cell development and subsequent antibody responses to a broad range of pathogens or vaccines. Aside from changes in the cytokine milieu, other mechanisms to nonspecifically activate T cells include the ability of some PAMPs to act as super-antigens, able to activate T cells via non-specific binding on the Vβ chain of TCRs. The rabies vaccine, whose nucleocapsid component possesses super-antigenic properties, could operate in this manner to produce NSEs (32). However, NSE mechanisms such as the bystander effect are unlikely to be the main mode of action in early life because infants lack pre-existing immunity to several microbes, not to mention their humoral responses are still not optimal (22, 33).

Besides bystander activation, the general elevation of cytokine levels, like the increased IFN-γ production by T cells during a primary cell-mediated immune response to BCG, could activate macrophages to boost phagocytic capabilities and thereby protect against secondary bacterial (i.e., pneumococcal) infections (Figure 1A) (34). Macrophage phenotyping, using Fluorescence Activated Cell Sorting, demonstrated in young adults that the activation status of macrophages can persist up to a year (29). While this classical cross-protective immune response may play an important role in the reduction of all-cause mortality in infants, it is a short-lived protection that wanes soon after the primary BCG stimulus is cleared from the body. Moreover, BCG-exposed nude mice were protected against heterologous pathogens like *S. mansoni*, indicating that there are also other T-independent, non-classical mechanisms at play.

### Trained Immunity

Indeed, despite decades of research indicating the benefits of BCG, the biological processes behind both NSEs and *Mycobacterium*-specific protection are still an enigma (22). A recent immunological paradigm shift sheds light on the black box that is responsible for BCG NSEs, and supports the notion of a T- and Ab-independent mode of action for protection. The dogma has always been that innate immunity lacked immunological memory, while the opposite was true for adaptive immune responses. Recent discoveries in plants, invertebrates and mammals support the notion that innate immune cells do indeed display intrinsic memory characteristics. Such traits have been identified so far in macrophages, monocytes, NK, and other innate lymphoid cells (ILCs) like ILC2s (35). It is of interest to note that NK cells display both antigen-specific memory (as mentioned above) and non-specific memory traits more typical to innate immune cells. Meanwhile, allergen-primed ILC2s, unlike their Th2 cell counterpart, do not have antigen-specific receptors, but respond more strongly to changes in the cytokine environment upon secondary stimulation (like the bystander effect). ILCs demonstrate innate-like memory by responding to IL-33 induction rather than to a specific antigen, resulting in increased proliferation, and IL-5 and IL-13 production (36).

This memory-like phenomenon of innate cells, also referred to as trained immunity, received its namesake from the concept that innate cells encountering a vaccine or microbial component can be influenced or “trained” by a primary stimulus to improve responsiveness to an unrelated secondary stimulus. The development of an *in vitro* experimental model for trained immunity in primary monocytes paved the way to explore this groundbreaking concept in human cells. The technique has been optimized to investigate the ability of commonly studied stimuli, like BCG, to induce innate immune memory-like response in human monocytes (37). The protocol involves a primary stimulation with BCG to “train” monocytes isolated from healthy donor blood, followed by a resting period for the cells, and then re-stimulation with a stimulus like lipopolysaccharide (LPS), which mimics a secondary heterologous challenge (37). BCG immunization prior to collection of primary cells could allow for *ex vivo* studies as well. At the end of the protocol, cells from either *in vitro* or *ex vivo* approaches can then be harvested and examined for changes in epigenetic traits, proliferation, cytokine production, and metabolic characteristics such as glycolysis or induction of autophagy.

Currently, the proposed mechanism for trained immunity involves the epigenetic reprogramming of innate immune cells, like monocytes, that have been activated by a microbial or vaccine component, and subsequently induced to produce broadly acting cytokines (i.e., TNFα, IL-6, IL-1β, IL-10). Depending on the nature of the primary stimulus, it can allow for the activation of transcription factors (TFs) like NFκB, AP-1, and STATs in myeloid cells, that bind to enhancers and promoters of pro-inflammatory or anti-inflammatory genes, downregulating some of them while upregulating others (38). The TFs allow for the recruitment of RNA polymerase and chromatin modifiers to regulate gene expression (Figure 1B). This phenomenon could lead to the creation of latent or “*de novo*” enhancers, which are genetic regulatory elements that acquire enhancer-like epigenetic modifications in response to specific stimuli (Figure 1B). Even, when the immune response clears the primary stimulus, some of these more stable modifications (i.e., methylation or acetylation) remain. As a result of their upgraded epigenetic status, innate immune cells are able to respond more vigorously to a secondary stimulus (Figure 1B). Immunization with BCG has also been shown to induce epigenetic changes in human monocytes *ex vivo* (35).

Another way to explore human trained immunity by BCG was recently tested, where both primary and secondary stimulation of the immune system are done *in vivo* with BCG and the yellow fever vaccine (YFV). Yellow fever vaccine is a rare opportunity to perform “challenge studies” in humans, since it is a live attenuated vaccine already in human use with detectable, post-vaccination viremia (39). While BCG-induced trained immunity was able to reduce YFV viremia, it did not seem to interfere with the efficacy of the second vaccine, as YFV-induced humoral responses were unaltered by BCG. These results thus support the clinical relevance of BCG trained immunity; it protects against a secondary viral stimulus without interfering with adaptive mechanisms that are also in play (39).

In the case of the YFV “challenge” model, IL-1β was noted to be an especially important cytokine to achieve a more robust innate immune response against the secondary YFV insult following BCG vaccination (39). Its role as a mediator in monocyte trained immunity was supported by both epigenetics and immunological methods. Chromatin immunoprecipitation sequencing (ChIP-seq) was used to detect genome-wide changes in the distribution of histone markers denoting enhancers or active promoters, particularly acetylation on histone 3 lysine 27 (H3K27ac); these activation marks were upregulated in the regulatory elements of pro-inflammatory cytokine genes, such as IL-1β, IL-6, and TNFα. In line with previous findings, genes for Akt/mTOR (mammalian target of rapamycin) kinases and NOD2 (nucleotide-binding oligomerization domain-containing 2) were also epigenetically marked and corresponded to the upregulation in cytokine production *ex vivo*, measured via ELISA. With viral challenge upon administration of YFV, individuals vaccinated with BCG a month earlier exhibited lower YFV viremia. Increased IL-1β levels, in particular, correlated with reduced YFV viremia (25, 39). This challenge model paves the way for future studies that must further investigate mechanisms of trained immunity, which were first identified using *in vitro* and *ex vivo* approaches, in a clinical context and confirm these results.

**Figure 2:**
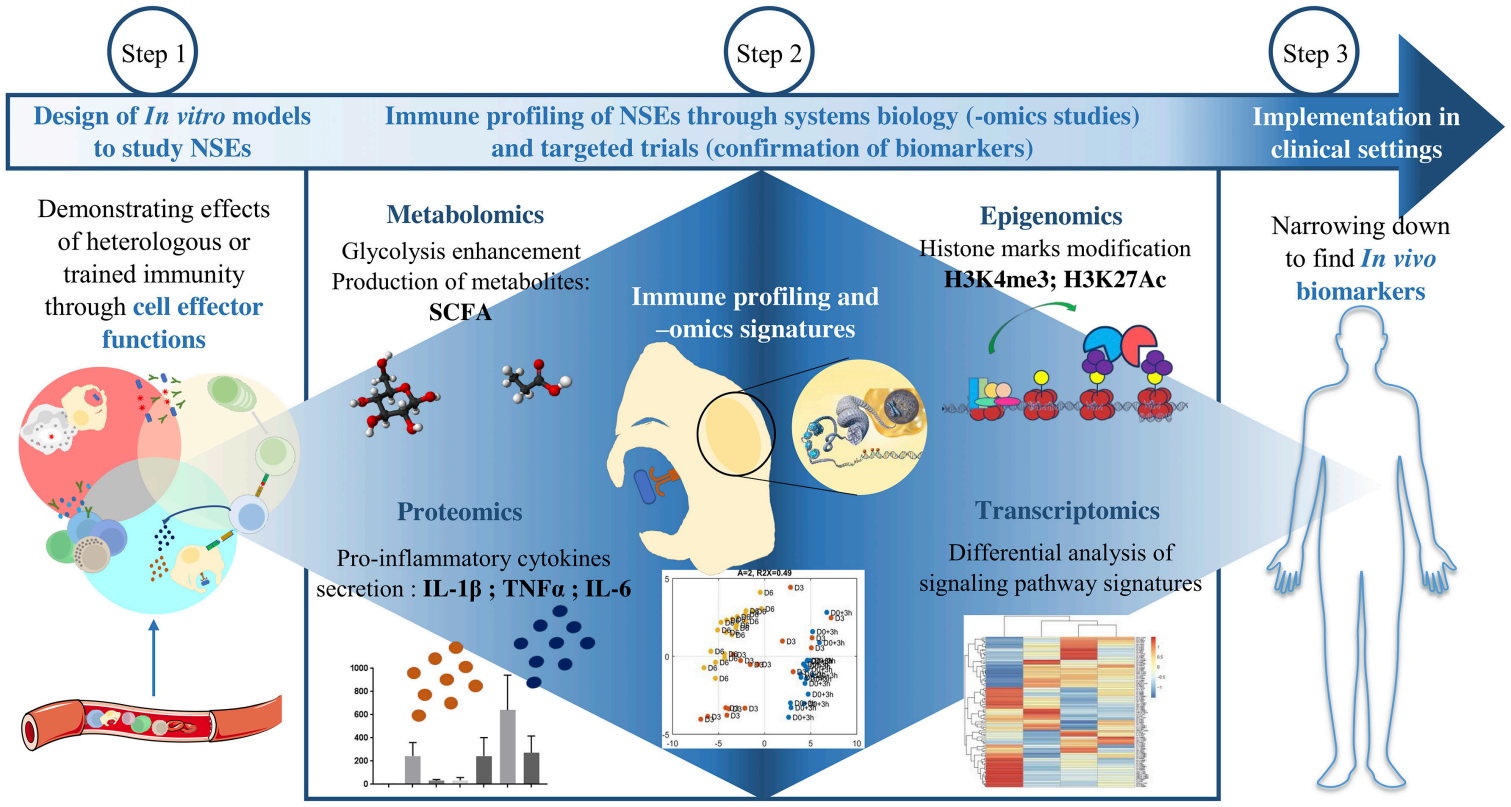
Approaches to investigate BCG NSEs. After designing a model to study trained innate immunity *in vitro*, -omics studies help to identify the global cellular pathways involved. The variations in gene expression are assessed both at the transcriptomic level and at the epigenetic level and then confirmed at the protein level. The modifications of cell metabolism and effector functions are also various angles by which to grant NSEs biological plausibility. The last step consists in narrowing down these modifications in order to find protective biomarkers which can be applied *in vivo* in clinical settings.

These findings also demonstrate a classic case of how “-omics” technologies have revolutionized the study of vaccine NSEs, providing a “global” view of changes that cells undergo upon receiving external signals from their environment, whether it be at the genomic, epigenomic, transcriptomic, metabolomic, or proteomic level (Figure 2) (1). In this case, genome-wide epigenetic studies have greatly expanded the capacity to identify molecular pathways that may have a key role in monocyte training after BCG immunization, such as proinflammatory cytokine induction, NOD2 pattern recognition receptor (PRR) signaling, and upregulation of the Akt/mTOR signaling pathway that regulates glycolysis (39). Performing -omics studies on cells trained *in vitro* provides insight on potential biomarkers associated to protection.

Other studies also employed epigenetics to elucidate the role of immunometabolism in trained immunity (40, 41). ChIP analysis of BCG-stimulated monocytes revealed more H3K4me3 and less H3K9me3, markers of active euchromatin and suppressive heterochromatin respectively, on promoters of glycolysis genes. Downstream effects of these epigenetic changes on transcription were confirmed via quantitative reverse transcription polymerase chain reaction (RT-qPCR). A similar pattern was seen on the *IL6* and *TNFA* promoters. Notably, the marks on these pro-inflammatory cytokine genes returned to baseline when inhibitors of glycolysis were used, like rapamycin. Therefore, while BCG-induced training is required to alter cell metabolism, these metabolic changes in turn seem to be necessary to maintain a trained phenotype (41).

Metabolic characterization of trained cells was thus performed as follow-up. Glycolytic rate was determined by assessing the glucose consumption from cell culture supernatant, lactate production (the end-product of glycolysis) was measured as a function of the extracellular acidification rate and oxidative phosphorylation was detected via the cells' oxygen consumption rate using the Seahorse Cell Mito Stress Test Kit and other fluorometric assays (41). Pro-inflammatory (M1) macrophages have a tendency toward increased glycolysis and lactate production, while anti-inflammatory (M2) macrophages appear to depend more on oxidative phosphorylation and lipid metabolism. Similarly, monocytes and macrophages trained with BCG shift to an M1-like phenotype (Figure 1B). Glucose consumption increased upon secondary stimulation of these cells, mirrored by a proportional increase in lactate. Notably, the ensuing induction in pro-inflammatory cytokines upon a secondary stimulus was abrogated when blocking the Akt/mTOR pathway during BCG training.

Growing evidence proposes that BCG training and subsequent metabolic changes fuel each other in a regulatory loop. Mevalonate (an intermediate in the cholesterol synthesis pathway) and fumarate (involved in glutamine metabolism), are measurable metabolites that regulate trained immunity through enhanced Akt/mTOR signaling by promoting signaling through the insulin-like growth factor 1 (IGF1) receptor and regulating chromatin methylation, respectively (Figure 1B) (42). The exact links connecting metabolic changes to trained immunity are still under investigation. It has been described that the enhancement of glycolysis led to an accumulation of Krebs cycle substrates such as fumarate or succinate. These metabolites were shown to stabilize HIF-1α (hypoxia-inducible factor 1α), a TF downstream of Akt/mTOR signaling that upregulates glycolytic genes and IL-1β production (Figure 1B). Fumarate has also been described in the inhibition of histone demethylases, namely KDM5 (43).Lactate, which is the end product of glycolysis has also been demonstrated to inhibit histone deacetylase (HDAC). Together these two observations suggest that shifting the metabolic balance toward glycolysis will increase the proportion of some metabolites which in turn will bring the necessary chemical groups to chromatin-modifying enzymes and thereby enable epigenetic modifications associated with trained immune features. Glycolysis inhibition studies were also performed and resulted in an impairment of trained immunity induced by BCG or other stimulants like β-glucans. Furthermore, direct fumarate supplementation led to β-glucan-like transcriptional profiles of monocytes although fumarate alone was not able to reproduce all the modifications implicated in the trained immunity profile. Moreover, increasing glycolytic flux will also fuels lipogenic pathways such as cholesterol biosynthesis of which mevalonate is an intermediate metabolite. Mevalonate, an intermediate metabolite in the cholesterol biosynthesis pathway, has been described as a key regulatory factor in BCG-stimulated trained immunity as the inhibition of its biosynthesis through statins impaired training of monocytes *in vitro* (44).The extreme intricacy of metabolic shifts and cellular functional responses is due to the high number of interconnected pathways regulated by positive and negative feedback loops (itaconate pathway for instance) (45). The precise role and causality of these molecular and cellular networks will be further investigated in the coming years in order to better understand immunometabolism in the context of trained innate immunity.

At least in the case of BCG, *in vitro* models also demonstrated that autophagy of these cells is a vital component of trained immunity. Independently, NOD2 stimulation with PAMPs was also shown to be required (25). Autophagosomes may thus help to process PAMPs, such as muramyl dipeptide from BCG, to be recognized by the NOD2 intracellular PRR (Figure 1B). This event would lead to a downstream signaling cascade and induction of epigenetic changes, such as the H3K4me3 that was previously described to upregulate pro-inflammatory genes (Figure 1B) (25, 46). Suboptimal induction of autophagy via drug inhibitors and certain autophagy-related gene polymorphisms led to the absence of epigenetic modifications seen after BCG stimulation (47). Autophagy activity was assessed through measuring levels of mediators such as autophagy-related proteins LC3A and LC3B, that are involved in autophagosome formation and may be useful as biomarkers of trained immunity by BCG (47).

It was recently demonstrated that BCG can act even in the early stages of myelopoiesis and instruct hematopoietic stem and progenitor cells (HSPCs) in the bone marrow to develop into monocytes and macrophages with a specific epigenetic program. Training of HSPCs may be a vital part of vaccine NSEs, influencing mature myeloid cell function in peripheral organs (48, 49). Both epidemiological and proof-of-concept *in vitro* studies demonstrated that the trained phenotype persists in human monocytes from 3 to 12 months following BCG immunization, in contrast to the usual lifespan of circulating monocytes of up to a day (29, 35).

### BCG-Induced Immune Responses in Favor of NSEs

Overall, *in vitro* models and *ex vivo* studies of human immune cells help conceive a general scheme of the immune response in individuals vaccinated with BCG and how it relates to trained immunity, as well as other NSE mechanisms. On the innate arm of the immune response, NK cells, and monocytes undergo epigenetic reprogramming, with an upregulation of permissive H3K4me3 and downregulation of inhibitory H3K9me3 histone marks on the genes of pro-inflammatory cytokines and regulators of glycolysis, among other targets in the genome. Muramyl dipeptide of BCG may have a vital role to play in inducing these changes, acting as a ligand for NOD2-dependent PRR signaling in monocytes and NK cells. Using an *in vitro* model of trained immunity, it was demonstrated that human peripheral blood mononuclear cells (PBMCs) trained with BCG resulted in only minor changes in cell size and morphology, but produced a 4-5-fold increase in IL-6 and TNF-α in response to secondary challenges with LPS or the synthetic lipopeptide Pam3Cys, 6 days later (37, 50). PBMCs isolated from BCG-immunized adults also produced elevated TNF-α and IL-1β levels after secondary challenge 3 months post-vaccination with sonicated *Mycobacterium tuberculosis* or heterologous pathogens such as heat-killed *S. aureus* or *C. albicans* (29).

The heightened capacity to produce innate pro-inflammatory cytokines was even observed after secondary challenge with *E. coli* LPS in monocytes isolated 1 year after immunization. These one-year post-vaccination monocytes expressed elevated levels of activation markers such as CD14, CD11b, TLR4, and mannose receptors, which suggest that BCG-mediated NSEs may be the result of long-term changes in innate immune cell phenotype that allow for non-specific protection against heterologous pathogens (29, 50). In contrast, the heightened expression of activation markers and pro-inflammatory cytokines in response to heterologous challenge was not observed in monocytes isolated from unvaccinated individuals (29, 50).

Maintenance of long-term NSEs induced by BCG may also depend on adaptive mechanisms, in addition to trained immunity. Although, innate immune activation markers were elevated against LPS secondary challenge 1 year post-vaccination, this was not the case for other non-Mycobacterial challenges, such as *C. albicans* and *S. aureus*, where the immune response resembled that which would be mounted against a primary infection. BCG-induced bystander activation of CD4+ T cells can complement trained immunity to prolong NSEs. In the same study by Kleinnijenhuis et al. Th1 and Th17 cytokine (IFN-γ, IL-17, IL-22) levels remained elevated in PBMCs 1 year after BCG immunization, regardless of which secondary non-Mycobacterial stimuli was provided. Unlike these heterologous cell-mediated responses, non-specific antibody responses induced by BCG vaccination are underexamined. Some studies suggest that BCG immunization at birth contributes to humoral responses from subsequent vaccination with *Haemophilus Influenzae* type b, pertussis, hepatitis B and pneumococcal antigens, but the duration and extent of protection remain controversial due to differences in countries' vaccination schedules and the BCG strain used (51–53). Therefore, further investigation into Ab-mediated NSEs with comparable study designs need to be conducted, especially considering that other studies demonstrate a different outcome, where BCG immunization has no impact or a negative effect on Ab production to other vaccines (34, 53).

In addition, it is also important to consider that trained immunity has mainly been characterized in adult monocytes. As in adult *ex vivo* studies, CD4+ T cell proliferation and cytokine responses appear to be enhanced in infant monocytes *ex vivo*, as infants vaccinated with BCG at early time points (0–2 months old) demonstrated elevated levels of lymphocyte proliferation and Th1/Th2 cytokines compared to unvaccinated infants or those vaccinated at later time points (20, 51, 53, 54). As with potential BCG-induced humoral NSEs, the extent of BCG-induced trained immunity in infants remains controversial and varies with each study's host country, their extended programs of immunization and the immunogenicity of the strain of BCG used. For example, elevated monocyte cell surface activation markers were not observed after heterologous challenge with LPS, synthetic lipopeptide Pam3CSK4, *C. albicans* or *S. aureus* on whole blood isolated from infants immunized with BCG at 6 weeks (30). However, in this study, challenge with Pam3CSK4 did indeed result in a significant upregulation of the CD69 activation marker on NK cells, suggesting NK cells may play a role in BCG NSEs, as in adults. Another study closely resembled adult studies, where whole blood samples from low-birth-weight infants vaccinated with BCG at birth in Guinea-Bissau were challenged with Pam3CSK4 a month later. Elevated levels of IL-1β, TNF-α, IL-6, and IFN-γ were observed (20).

One could therefore observe some promising results of innate immune response potentiation and elevated CD4+ T cell responses in favor of BCG NSEs in both infants and adults. A complex interplay between epigenetic reprogramming, cell metabolism and innate immune machinery enables BCG-trained cells to respond more robustly against a second heterologous insult. Complementing innate immune cell potentiation is the potential for BCG to induce heterologous T cell responses in vaccinated individuals. The interplay between innate and adaptive immune mechanisms could be responsible for the long-term NSEs that in some cases have persisted years after vaccination; while trained immunity may play a larger role in NSEs in early life, adaptive mechanisms can maintain NSEs even when training immunity starts waning (53). However, the duration of BCG-induced trained immunity in infants is still unclear and requires further investigation. To better characterize BCG NSEs, it is important to identify which of the described cellular changes can be used as biomarkers of protection, and then employ them in clinical studies that supplement epidemiological studies monitoring all-cause mortality (Figure 2). Finally, biomarkers should be standardized and thresholds should be made, so that results between studies can be compared. Following the systematic review done by the SAGE Working Group, improved study designs to investigate BCG NSEs are being used as a means to achieve these objectives.

## Toward the Future: New and Improved Studies

As basic science uncovers the mysteries behind BCG's mechanisms of action and proposes how the vaccine can induce non-specific protective effects, it is now important to apply these findings to explain the reduction in all-cause mortality observed in many epidemiological studies. Given the laboratory findings thus far, trained immunity, in combination with adaptive mechanisms, likely have a role to play in BCG NSEs. In order to produce stronger evidence of the heterologous benefits of BCG at a population level, more recent epidemiological studies aim to characterize differences in the immune response of BCG-vaccinated vs. unvaccinated individuals.

Prior to a decade ago, the only randomized trials, as well as a large portion of observational cohort studies, were conducted in Guinea-Bissau and other areas of West Africa. Meanwhile, the quasi-randomized studies performed in North America over 50 years ago were the only studies done in high income countries that were reviewed by the SAGE NSE Working Group from 2012 to 2014, when they conducted a systematic review of epidemiological data that may support the impact of BCG and other vaccines on infant all-cause mortality. The Working Group acknowledged that high mortality settings like Guinea-Bissau are often required for mortality studies, in order to obtain an adequate study power and detect relative effects. On the other hand, in these countries it is difficult to draw inferences due to the difficulty of controlling for all of the confounders affecting mortality (11). Recent studies conducted in both high income and low mortality, as well as low income and high mortality, settings aimed to counter this issue raised by the SAGE (Table 2).

**Table 2:** Recent studies on BCG NSEs following the SAGE working group's systematic review on vaccine NSEs to associate the reduction in all-cause mortality with immunological outcomes.

**Country (study period)**	**Type of study**	**Subject follow-up period**	**% Reduction in all-cause mortality or morbidity (95% CI)**	**Testing for NSEs**	**References**
Denmark (2012-2015)	Randomized	15 months	No significant reduction	Potentiating effect of BCG on cytokine production (TNF-α, IL-6, IL-10)	(55, 56)
Uganda (2015-)	Randomized	10 weeks	Unpublished	IL-1β, IL-6, IL-10, TNF-α, and IFN-γ cytokine levels after secondary stimulation. H3K4me3 on cytokine genes in peripheral blood monocytes.	(57) (results on mortality unpublished)
Guinea-Bissau (2008-2013)	Randomized	12 months	30% (−4; 53)	Measure increases in responses to heterologous stimulation (elevated IL-1β, IL-6, TNF-α, and IFN-γ response)	(20, 58)
Spain (2015)	Retrospective cohort	N/A	41.4% (40.3; 42.5)	None	(59)

For example, the Danish Calmette study, running from 2012 to 2015, randomized over 4,000 infants into those vaccinated with BCG or those who followed the normal Danish vaccination schedule and did not receive BCG. However, this study did not show any added effect of BCG on all-cause hospitalization (55, 56). Meanwhile, a retrospective cohort study looking back to hospitalization rates in Spain from 1997 to 2011, using the Official Spanish Registry of Hospitalizations, favored the opposite outcome; a randomized clinical trial in Uganda monitored clinical illness in 560 (either BCG-vaccinated or non-vaccinated) neonates and demonstrated similar results to those in Spain (Table 2) (57, 59). Varying results may be due to study design, variable strains of BCG, the sample population, and other unknowns. To eliminate as many confounders as possible that may influence the ability to detect BCG NSEs, it is no wonder that the SAGE demands that studies be done in more diverse populations, yet in more controlled settings.

Moreover, the Working Group's evaluation from 2012 to 2014 ascertained that although epidemiological studies up until then examined all-cause mortality in relation to BCG, they failed to examine in any depth the mechanisms behind the reduction in mortality. However, they admit that death by TB is secondary in infants compared to sepsis, pneumonia, and diarrhea, so the significant impact that BCG has on all-cause mortality is unlikely to stem only from TB-specific protection (11). Studies are now trying to identify immunological endpoints during clinical studies that may explain NSEs in the population. A more recent randomized trial in Guinea-Bissau not only monitored all-cause mortality of low-birth-weight infants, but also performed whole-blood assays to measure cytokine levels in response to heterologous secondary challenge after BCG vaccination (Table 2). Results were promising; elevated levels of IL-1β, IL-6, TNF-α, and IFN-γ following *ex vivo* secondary heterologous challenges of whole blood from BCG-vaccinated infants, compared to unvaccinated infants mirrored a 30% reduction in the neonatal mortality rate (20, 58).

## NSEs From Other Vaccines

After discussing the underlying mechanisms of BCG NSEs, it is important to note that NSEs have been studied in other vaccines as well. As mentioned earlier, the epidemiology of measles-containing vaccines and OPV have also been analyzed for the presence of NSEs. Measles-based vaccines have been observed to greatly reduce child mortality in low income communities in Haiti (3, 60). In the case of a vaccine targeting measles, a reduction in all-cause mortality would seem logical, given that the measles virus itself induces transient immunosuppression in infected individuals; immunization is postulated to prevent ablation of the previously existing memory T and B cell repertoire within an individual caused by measles virus infection (61). However, certain observations suggest that other mechanisms besides protection from measles virus-induced immunosuppression are responsible for these NSEs. For instance, a reduction in morbidity is observed in regions where the measles virus has already been eliminated, not to mention an increase in NSEs is observed even in the presence of maternally derived antibodies that could interfere with the efficacy of the live vaccine (62, 63).

While live attenuated vaccines in humans appear to have non-specific protective effects, the same has yet to be observed with killed or subunit vaccines. In fact, the opposite effect could be induced; Diphtheria-Tetanus-Pertussis (DTP)-vaccination of children, especially girls, has resulted in an increase in all-cause mortality, notably when administered simultaneously or before live attenuated vaccines such as measles-containing vaccines (64). The global analysis conducted by the SAGE Working Group, which excluded studies with poorly defined controls, led to firmer evidence that there may indeed by an increase in all-cause mortality in DTP-vaccinated children, mostly observed in Guinea-Bissau (1, 11). Therefore, while vaccines like BCG and measles exhibit potentially beneficial NSEs that reduce all-cause mortality, the reverse can also be true, where vaccine NSEs could pose as a threat to public health. There is thus a dire need to continue with randomized trials, resolve contradictions between the various studies of vaccine NSEs and produce more convincing evidence for their existence, such that public health authorities can confidently act on the information.

Moreover, the role of NSEs on public health could be expanded further beyond its impact on child mortality, which was the focus of the BCG, measles and DTP epidemiological studies presented above. In fact, NSEs could even exist outside the scope of human vaccines, as observations have also been noted in the veterinary field. The rabies vaccine, like BCG in humans, was also observed to reduce all-cause mortality in dogs via non-specific protection (32). In a low-income region of South Africa, an observational study was conducted from 2012 to 2015 in owned, free-roaming dogs to study the impact of rabies vaccination. Vaccination against rabies is provided free annually in South Africa as a disease control measure, rendering the region a good setting to study the impact of the vaccine; public vaccination campaigns in part reduce the bias that the NSEs observed in rabies-vaccinated dogs may be attributed to a generally better quality of life with more nutrition and visits to the vet compared to unvaccinated dogs. Overall, these observations must still be validated with an improved study design, through the implementation of randomized controlled clinical studies, the elimination of confounding factors such as owner behavior and accessibility of veterinary care, and segregating reduction in mortality due to rabies and similar lyssaviruses from heterologous etiological agents. Unlike the case with human vaccines, rabies is a killed vaccine that appears to have protective NSEs.

## Conclusion

In conclusion, heterologous effects of vaccines are coming to be realized as a real phenomenon by immunologists and epidemiologists, with mounting evidence suggesting biological plausibility. While the WHO's current position on the matter asserts that there is poor and biased evidence supporting vaccine NSEs, they do acknowledge the high plausibility that NSEs may be one of the puppeteers behind the triumphant story of vaccines. Thus, they call for the need to further solidify a biological basis and better understand the public health impact, such as through determining a specific vaccination schedule and the resulting proportion of deaths that would actually be prevented in each population (11).

As future vaccine studies evolve to meet these expectations, NSEs have the potential to influence the current WHO recommendations on childhood vaccination. Policy changes would not be made based on the question of whether vaccines should continue to be used universally or not, but to influence the timing, sequence or co-administration of these vaccines according to the existence of both protective and harmful NSEs affecting general childhood death rates (11). Recommendations would therefore also address the use of vaccines whose target disease is already eliminated, but may confer non-specific protection to other diseases (46).

The universal acceptance of vaccine NSEs would advance more easily once supranational organizations like the WHO move in favor of their existence. In the case that a new, more efficacious vaccine against TB is developed, it will become important to determine whether this vaccine can also confer NSEs, and whether BCG should still remain in use due to its reduction in all-cause mortality in early life. Vaccination policies need be restructured to provide the maximum benefit, both specific and non-specific. For example, observational studies regarding DTP NSEs clash; some groups suggest DTP is beneficial while others demonstrate it has deleterious heterologous effects on all-cause mortality, such as through increased incidence of rotavirus infection in girls (11, 65). Although, the SAGE judged it as insufficient evidence to endorse a policy change, results from Bangladesh, India, and Senegal contradict the current WHO recommended schedule of administering DTP after BCG, and instead imply that simultaneous administration of these vaccines may reduce deleterious effects and lower mortality (12).

The incorporation of NSEs into vaccinology would also greatly impact vaccine design; new vaccine technologies can aim to prime both non-specific and pathogen-specific immunity. Many adjuvants already have innate immune targets, and perhaps NSEs can provide enlightenment on their mode of action. Primary endpoints for vaccine efficacy trials may now include innate immune markers as correlates of protection, in addition to adaptive markers like antibody titers or IFN-γ. In addition, the trained immunity concept adds a new chapter to macrophage biology. Previously, depending on the immunological context, such as during infection or in the tumor microenvironment, macrophages are delineated into subsets like “M1” and “M2,” or myeloid-derived suppressor cells, without any universal classification for these cells that can span across various disciplines. More recently, a collective framework to describe activated macrophages was proposed, based on cell markers, how these cells were isolated, and the immunostimulants used to activate them (66). For example, “M1” and “M2” could now be referred to as M(IFN-γ) and M(IL-4), based on their activators. Once the trained immunity concept gains ground, it would be interesting to see how it fits into this grander scheme of macrophage nomenclature, taking into account how trained immunity can integrate the various terminologies used across studies. Trained immunity may also have important implications in countering immuno-senescence, where traditional adaptive immunity wanes in the elderly (46). As is the case with BCG being an effective therapy against bladder cancer, NSEs of vaccines can extend beyond protection against infectious diseases; by broadly manipulating the immune system, these vaccines can serve as therapeutics against other ailments, such as cancer and immunodeficiency. The reverse is also true, though; while NSEs can ameliorate immunodeficiency, they can also potentiate autoimmune conditions.

Innovative vaccine designs, that take advantage of NSEs, can also be applied to the “One World One Health” initiative. Protecting animal companions from maladies is an improvement to the overall public health of society, both by reducing health care costs for animals and by decreasing the incidence of zoonotic disease transmission as is the case with rabies (67). The use of such public health measures could also serve as an alternative to antibiotic use to protect production animals against infectious diseases (68, 69). Furthermore, vaccines that can provide early and broad protection via trained immunity would greatly benefit the agricultural industry, where for instance many chicks, piglets and calves are lost each year due to the inefficacy of many vaccines in very early life. Factors such as maternal antibody interference, an immature adaptive immune system (which in mammals becomes apparent after weaning), or even the diversity of farming practices and environmental conditions are at play in this period of an animal's development.

Overall, the vaccine NSE concept has the potential to improve both human and veterinary vaccinology and provide new means to answer both immunological and public health questions. NSEs can provide new concepts to assess what were previously invisible influences on mortality, and also combat challenging diseases. Addressing the NSE question will provide new insights on mechanisms through which existing vaccines work and may influence future vaccine design. The knowledge gained throughout the investigation of NSEs could lead to modifications of global vaccination policies to optimize the benefits of vaccination in reducing childhood mortality and morbidity.

## Author Contributions

DU and KD conceived the outline of the manuscript. DU wrote the manuscript. DU, SP, and KD edited the figures of the manuscript. All authors contributed to manuscript revision, read and approved the submitted version.

### Conflict of Interest Statement

DU, SP, LC, HP, and KD were employed by company Boehringer Ingelheim Animal Health. The remaining author declares that the research was conducted in the absence of any commercial or financial relationships that could be construed as a potential conflict of interest.
